# Inorganic Fiber Lung Burden in Subjects with Occupational and/or Anthropogenic Environmental Asbestos Exposure in Broni (Pavia, Northern Italy): An SEM-EDS Study on Autoptic Samples

**DOI:** 10.3390/ijerph18042053

**Published:** 2021-02-19

**Authors:** Silvia Damiana Visonà, Silvana Capella, Sofia Bodini, Paola Borrelli, Simona Villani, Eleonora Crespi, Andrea Frontini, Claudio Colosio, Elena Belluso

**Affiliations:** 1 Unit of Legal Medicine and Forensic Sciences, Department of Public Health, Experimental and Forensic Medicine, University of Pavia, 27100 Pavia, Italy; sofia.bodini01@universitadipavia.it; 2 Department of Earth Sciences, University of Torino, 10125 Torino, Italy; silvana.capella@unito.it (S.C.); elena.belluso@unito.it (E.B.); 3 Interdepartmental Center for Studies on Asbestos and Other Toxic Particulates “G. Scansetti”, University of Torino, 10121 Torino, Italy; 4 Unit of Biostatistics and Clinical Epidemiology, Department of Public Health, Experimental and Forensic Medicine, University of Pavia, 27100 Pavia, Italy; paola.borrelli@unipv.it (P.B.); simona.villani@unipv.it (S.V.); 5 Laboratory of Biostatistics, Department of Medical, Oral and Biotechnological Sciences, University “G. d’Annunzio” Chieti-Pescara, 66100 Chieti, Italy; 6 Occupational Health Unit, Santi Paolo e Carlo Hospital, 20142 Milano, Italy; eleonora.crespi@asst-santipaolocarlo.it (E.C.); claudio.colosio@unimi.it (C.C.); 7 Department of Life and Environmental Science, Polytechnic University of Marche, 60131 Ancona, Italy; a.frontini@univpm.it; 8 Department of Health Sciences, University of Milan, 20122 Milano, Italy

**Keywords:** asbestos, asbestos bodies, malignant mesothelioma, lung fiber burden, SEM-EDS

## Abstract

Increased mortality due to malignant mesothelioma has been demonstrated by several epidemiologic studies in the area around Broni (a small town in Lombardy, northern Italy), where a factory producing asbestos cement was active between 1932 and 1993. Until now, the inorganic fiber burden in lungs has not been investigated in this population. The aim of this study is to assess the lung fiber burden in 72 individuals with previous occupational and/or anthropogenic environmental exposure to asbestos during the activity of an important asbestos cement factory. Inorganic fiber lung burden was assessed in autoptic samples taken from individuals deceased from asbestos-related diseases using a scanning electron microscope equipped with an energy-dispersive spectrometer. Significant differences in the detected amount of asbestos were pointed out among the three types of exposure. In most lung samples taken from patients who died of mesothelioma, very little asbestos (or, in some cases, no fibers) was found. Such subjects showed a significantly lower median amount of asbestos as compared to asbestosis. Almost no chrysotile was detected in the examined samples. Overall, crocidolite was the most represented asbestos, followed by amosite, tremolite/actinolite asbestos, and anthophyllite asbestos. There were significant differences in the amount of crocidolite and amosite fibers according to the kind of exposure. Overall, these findings provide novel insights into the link between asbestos exposure and mesothelioma, as well as the different impacts of the various types of asbestos on human health in relation to their different biopersistences in the lung microenvironment.

## 1. Introduction

Asbestos is the term for a family of naturally occurring fibrous minerals that are widespread all over the world. The word asbestos defines mineral species that occur as bundles of fibers and that can be separated into thin threads. They are classified as asbestos by World Health Organization (WHO) when they occur in a respirable size with certain dimensions (length > 5 µm, width < 3 µm, aspect ratio greater than or equal to 3:1 [1]). Six different minerals belong to the asbestos group: actinolite asbestos, tremolite asbestos, anthophyllite asbestos, grunerite asbestos (also called amosite from its commercial name, which is an acronym for Asbestos Mines of South Africa), crocidolite, and chrysotile (the first five are amphiboles, whereas chrysotile is a serpentine), according to the international nomenclature.

Even though the use of asbestos has been banned in European countries (in Italy in 1992, under law 257/1992), the widespread production and use of asbestos have caused unprecedented human suffering and still represent a major public health problem all over the world. Note that in many countries the mining and/or use of asbestos is still allowed (e.g., Russia, China, Kazakhstan, and Brazil). The latent onset of disease that occurs between about thirty and fifty years after exposure [2] has led to a catastrophic onslaught, still ongoing, as a result of people exposed decades ago.

There is a lack of recent studies that assess the inorganic lung content using analytical electron microscopy. This method represents the only way to quantify the inorganic fibers in lungs, as well as asbestos bodies (ABs), and, at the same time, to classify the fibers, according to their elemental composition, into mineralogical types. The paucity of such studies is due to the scarce availability of suitable samples. In fact, for the optimal execution of this technique, abundant samples of formalin-fixed normal lung parenchyma (free from neoplastic invasion and fibrosis) are required. The samples must be still in formalin (not paraffin-embedded). Moreover, the sampling site must always be the same, namely, the inferior lobe of the right lung. Obviously, the availability of such samples (and of suitable controls), mainly derived from autopsies, is limited. In fact, nowadays, autopsies in cases of malignant mesothelioma (MM) or other asbestos-related diseases are rarely performed.

Most studies on the inorganic lung content on postmortem samples failed to identify a correlation between asbestos concentration in lungs (and the concentration of each kind of asbestos) and MM [3,4,5,6,7]. On the contrary, other authors found a significantly higher concentration of asbestos fibers in MM compared to controls [8,9]. Another debated issue is the role of various kinds of fibers in determining MM. Even though crocidolite and amosite are recognized as the most hazardous types of industrial asbestos [10], lung content studies indicate that chrysotile has, indeed, an important role in MM causation [11,12]. Amphiboles are more represented, compared to chrysotile, in the lungs of MM patients [6,13]. On the whole, the papers about fiber burden analysis in lungs cited above do not suggest any significant correlation between MM and a particular kind of asbestos. Interestingly, studies about lung content provided proof that environmental exposure to asbestos (neighborhood and domestic) determines cumulative doses as high as those observed in some occupational exposure circumstances [14,15,16].

On the whole, the literature so far available about electron microscopy analysis of inorganic lung contents reveals very inconsistent conclusions about the link between the concentration of asbestos in lungs and the risk of developing MM. The studies summarized above, most of which are quite dated, were conducted using different methods of sample collection and preparation and different electron microscopy techniques (Transmission Electron Microscope—TEM, Scanning Electron Microscope—SEM, FEG SEM—Field Emission Gun SEM) with annexed Energy Dispersive Spectroscopy—EDS and instrumentations. On this basis, more research on a larger series of patients and controls is needed.

One of the many manufacturers in Italy that have represented important causes of asbestos exposure for both workers and the general population was an asbestos cement factory located in Broni (a small town in Northern Italy), active between 1932 and 1993, and producing asbestos cement artifacts using mixtures of commercial types of asbestos, mainly chrysotile and crocidolite, with smaller amounts of amosite [17]. People exposed to the above-mentioned source of asbestos, both occupationally and environmentally, who died and underwent a forensic autopsy are the subjects of the present study.

Inhabitants of the area of Broni and asbestos workers have been subjected to several epidemiological studies showing an increased mortality from MM [18,19,20,21,22,23]. Yet, the analysis of the asbestos lung content has never been performed on this population. This approach is essential in order to provide evidence about the effects of different concentrations of asbestos on human health, as well as the potential hazardousness of the various types of asbestos. Analytical electron microscopy investigation represents a fundamental and irreplaceable tool to obtain objective information about the inorganic fiber burden in lungs. Evaluations based only on epidemiological and anamnestic data are not always sufficient to estimate a subject’s exposure to asbestos during life and have been reported to sometimes produce misleading results [24]. With respect to what has been previously published about electron microscopy analysis of the inorganic fiber lung content, the present series provides novel insights because it includes individuals that were certainly exposed to asbestos (occupationally or environmentally), with all cases derived from the same factory and whose history is well documented. Moreover, the inorganic fibers contained in lungs were analyzed both in subjects who died from MM and in individuals who did not develop it, even though they were heavily exposed to asbestos (they died from asbestosis and its consequences). Indeed, there is a great need for data about asbestos amounts in MM patients (properly compared to exposed subjects without MM) assessed using electron microscopy, as most of the recent similar studies have investigated small series of subjects that died from MM [14,16,25].

The aim of the present study was to evaluate if inorganic fibers in human lungs, measured, classified, and quantified using a SEM-EDS, and especially the concentration of asbestos derived from anthropogenic environmental and/or occupational exposure that occurred during the activity of the factory, drive the occurrence of asbestos-related diseases differently.

## 2. Materials and Methods

### 2.1. Participants and Study Design

People who died of asbestos-related diseases confirmed by autopsy and histological examination were the target population. The eligible were all those died from MM and asbestosis, were living in a small town in Northern Italy (Broni, Pavia province)—where an important factory manufacturing asbestos cement was operating from 1932 to 1993—and were included in the records of the Forensic Medicine Department of the University of Pavia from 2000 to 2018. A total of 188 subjects were enrolled in the study. At the time of forensic autopsy, in each case, whole lungs were collected, formalin-fixed, and stored for further examination.

A retrospective cohort design was used. The study protocol was approved by the local Ethical Committee.

For the present study, a subsample of 72 subjects was selected from the cohort of those eligible using a non-proportional stratified random sampling by type of asbestos exposure.

### 2.2. Endpoints

The co-primary endpoints were the concentrations of total inorganic fibers, asbestos fibers, and ABs, as well as the concentrations of the various types of asbestos (chrysotile, crocidolite, amosite, tremolite/actinolite asbestos, and anthophyllite asbestos) in lung samples of the selected subjects. To compute these concentrations, the inorganic fibers and ABs contained in 0.25 g of the right wet lung (inferior lobe) were counted, measured, and analyzed using SEM-EDS, according to a protocol described by Belluso et al. [26].

Briefly, the method consisted of chemical digestion (using sodium hypochlorite) of 0.25 g of formalin-fixed lung parenchyma (to remove organic materials) and filtration of the suspension through a polycarbonate membrane (Millipore, Darmstadt, Germany) with a diameter of 25 mm and a pore size of 0.45 µm. Afterwards, the filter, dehydrated and pasted on a pin-stub using a carbon tape, was examined by SEM. The observation was performed on an area of 2 mm^2^ of the filter at 2000 M using backscattered electrons.

According to fiber definition [27], only particles with a length-to-width ratio > 3, a length > 5 µm, and a width < 3 µm were considered. ABs were also counted.

The fiber chemical composition was analyzed using an EDS, Oxford Inca Energy 200, equipped with an INCA X-act SDD detector (Oxford Instruments NanoAnalysis, Bucks, UK).

The numbers of detected inorganic fibers and ABs were normalized to 1 g of dry tissue, as indicated by international guidelines [28,29], reporting the concentration in terms of the burden of inorganic fibers, asbestos, and ABs per gram of dry lung tissue weight as ff/gdw.

To identify the different types of inorganic fibers, we compared the EDS spectra with a database internal to the laboratory.

Since the technique used here does not allow unequivocal identification of certain minerals having similar chemical composition and analogous morphology, it is not possible to distinguish chrysotile from asbestiform antigorite and tremolite asbestos from actinolite asbestos. Therefore, we used the following respective mineral group names: chrysotile/asbestiform antigorite, and tremolite/actinolite asbestos.

### 2.3. Control of Bias

The SEM-EDS investigation of the inorganic fiber lung burden was carried out in two laboratories. Samples were distributed equally between each one. In order to minimize possible bias due to different instruments and microscopists, before data collection started, a detailed protocol including the sample preparation and the data collection parameters was defined according to the procedure described by Belluso et al. [26]. In addition, a periodic inter-laboratory control was made, comparing images and spectra obtained, when the number of samples reached 10, 20, 40, 60, and 72. In addition, five samples were analyzed by both observers, revealing homogeneous measurements.

### 2.4. Variables

Anthropogenic environmental and occupational exposure information, the cause of death (investigated through a complete autopsy with histopathologic examination), and socio-demographic characteristics were extracted from the archives of the Forensic Medicine Department.

Regarding the type of exposure, in this paper we adopt the term “anthropogenic environmental exposure,” referring to people who lived in an area with air-dispersed asbestos from the asbestos cement plant [30,31]. The term “occupational exposure” refers to people who worked in the asbestos cement industry [32].

### 2.5. Statistical Analysis

Quantitative variables were summarized as the mean with standard deviation if normality was respected, and with the median, 25th, and 75th percentiles if not. To verify normality, the Shapiro–Wilk test was used. Three groups with different types of exposure were computed: occupational alone, anthropogenic environmental alone, and both. To evaluate differences in quantitative variables across groups of exposure to asbestos and histological type of MM, an analogous non-parametric test of analysis of variance (Kruskal–Wallis test) was applied, followed by the appropriate post-hoc test if significant. Bonferroni’s correction for multiple comparisons tests was applied. The evaluation of differences between subjects who died from MM and those who died from other causes was performed using a non-parametric unpaired t test (Mann–Whitney test). The relationships among quantitative variables were tested using Spearman’s correlation coefficient (rho). A *p*-value less than 0.05 was considered significant, apart from the post-hoc test in which, taking into account the correction for multiple comparison tests, the significance threshold was 0.0167 (*p*/k, assuming k = 3 contrast); however, in this case, the *p*-value was reported at the same scale and multiplied again for k. All analyses were performed using STATA 15^®^, StataCorp LLC., College Station, Texas, TX, USA

## 3. Results

We analyzed 72 cases, 86.1% of which were males. In total, 36.1% of the entire study group had anthropogenic environmental exposure to asbestos, 27.8% only occupational, and 36.1% had both.

Demographic data are summarized in Table 1.

In subjects with occupational exposure, the duration of asbestos exposure ranged from 6 to 480 months (median = 264, IQR 108–360 months), whereas in environmentally exposed individuals, the exposure was found to last between 36 and 720 months (median = 414, IQR 258–576). The latency (calculated only in MM cases), defined as the time elapsed between the beginning of exposure and the diagnosis, ranged between 16 and 60 years considering occupational exposure (median = 41 years, IQR 33–48) and 19–80 years (median = 53 years, IQR 42–65) considering anthropogenic environmental exposure. Note that some individuals lived in Broni, nearby the plant, for their entire lives.

The time elapsed between the end of exposure and death ranged between 8 and 44 years (median = 21 years, IQR 18–26).

In 81.9% of the analyzed cases, the cause of death, revealed by a forensic autopsy followed by histopathological examination and immunohistochemistry (using two positive and two negative markers, according to the guidelines [33]), was pleural MM. In particular, 65.5% of the deceased had epithelial MM, 10.3% sarcomatoid MM, and 24.1% biphasic MM. The diagnosis of asbestosis had been made during life according to international guidelines [33] and it was confirmed histopathologically (according to the typical morphologic aspect of the lungs as well as the presence of asbestos bodies under optical microscope observation). The survival time since diagnosis ranged between 1 and 379 months (median = 15 months, IQR 9.5–28.5).

### 3.1. Concentration of Inorganic Fibers, Asbestos, and ABs by Type of Exposure

**Figure 1:**
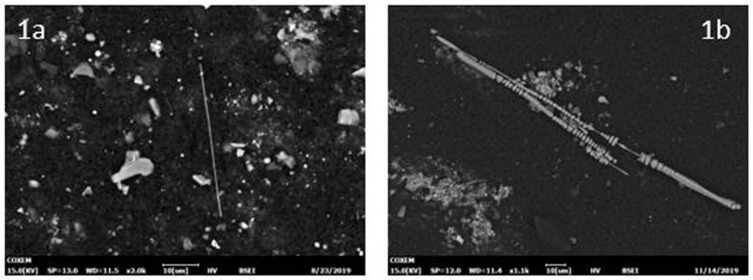
An example of an SEM (scanning electron microscopy) image (backscattered electrons) of an uncoated asbestos fiber (amphibole) (**a**) and of ABs (**b**).

Overall, the concentration of total inorganic fibers ranged from 0 ff/gdw to 6,679,195 ff/gdw (median = 62,928.8, IQR 13,801.8–253,703,5); the concentration of asbestos ranged from 0 ff/gdw to 5,689,685 ff/gdw (median = 24,199.7, IQR 0.0–167,984.5), and the concentration of ABs ranged from 0 ff/gdw to 3,003,538 ff/gdw (median = 6292.0, IQR 0–62,459.3). In most samples (77.8%), the concentration of uncoated fibers (Figure 1a) was higher compared to the ABs (Figure 1b), with a fiber/AB ratio ranging from 0.008 to 157.

Generally, a significant correlation between the amount of asbestos fibers and ABs was detected (Spearman’s rho = 0.471; *p* < 0.0001). However, the ratio between asbestos fibers and ABs was extremely variable, ranging from 0.0085 to 157. In 22.2% of the examined lung samples, only uncoated asbestos fibers (without any ABs) were detected. On the contrary, zero fibers and a number of ABs were detected in only four samples (5.5%).

In 19.4% of the examined lung samples, neither fibers nor ABs were observed; eight of them had only environmental exposure, three occupational exposure, and three of them had both kinds of exposure.

**Figure 2:**
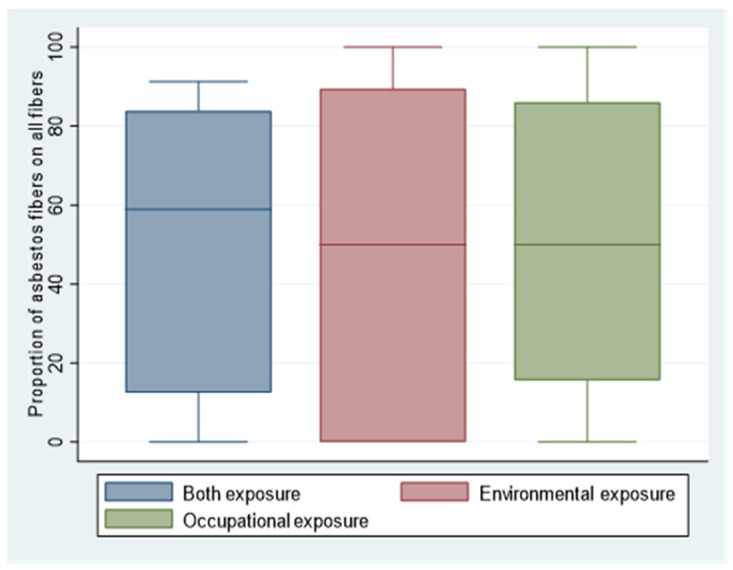
The proportion of asbestos fibers and other inorganic fibers (not asbestos classified), counted and classified using SEM-EDS, contained in lungs of individuals belonging to the three considered categories of asbestos exposure, represented as a median proportion.

Not only were asbestos fibers detected, but a considerable amount of other inorganic fibers were too. The proportion of inorganic fibers classified as not asbestos, compared to asbestos, was not significantly different according to the kind of exposure (*p* = 0.987). In individuals exposed only occupationally, considering the median, asbestos was 50% of the total inorganic fibers. In subjects exposed only environmentally, asbestos was 50% of the total inorganic fibers. In both exposures, the percentage of asbestos was the highest (59% of total fibers) (Figure 2).

We searched for differences in the amount of asbestos and ABs per ff/gdw among the three types of exposure (Table 2).

The only statistically significant result concerning this point regarded the concentration of ABs, which was different according to the kind of exposure. In particular, the amount of ABs was significantly higher in individuals with both exposures compared to those exposed only environmentally (*p* = 0.001). Unexpectedly, the amount of asbestos fibers was not significantly different across the three groups of exposure; in other words, individuals who worked in contact with asbestos had similar concentrations of asbestos in their lungs to subjects who lived nearby the plant.

### 3.2. Concentration of Each Kind of Asbestos by Type of Exposure

**Figure 3:**
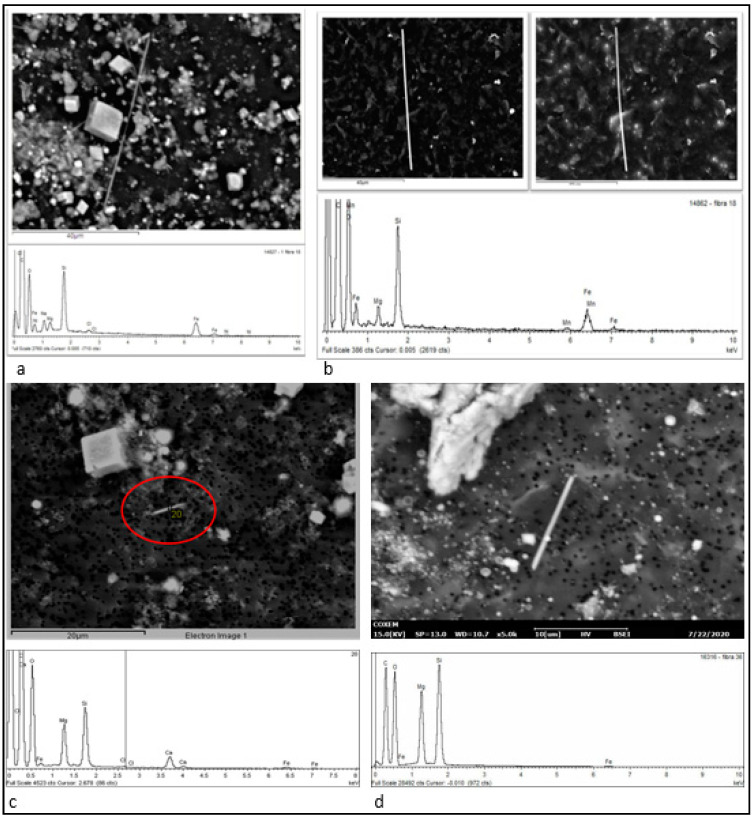
Some examples of SEM images and EDS spectra of each detected kind of asbestos. (**a**) Backscattered electron SEM image and EDS spectrum of crocidolite. (**b**) Backscattered (left) and secondary (right) electron SEM image, and EDS spectrum of amosite. (**c**) Backscattered electron SEM image and EDS spectrum of tremolite/actinolite asbestos. The cubic particles are NaCl residuals. (**d**) Backscattered electron SEM image and EDS spectrum of anthophyllite asbestos.

The asbestos fibers were classified on the basis of their shape, dimensional characteristics, and EDS spectrum, as follows, according to international guidelines and as specified in Section 2.2. Endpoints paragraph: (1) crocidolite; (2) amosite; (3) anthophyllite asbestos; (4) tremolite/actinolite asbestos (Figure 3); (5) chrysotile/asbestiform antigorite.

Tremolite asbestos and actinolite asbestos, similarly for chrysotile and asbestiform antigorite, were classified together because they could not be differentiated based on the EDS spectrum as they have similar chemical compositions. Tremolite/actinolite asbestos are both non-commercial asbestos, whereas the chrysotile/asbestiform antigorite group includes chrysotile (classified as asbestos, widely used to produce asbestos products) and asbestiform antigorite (not belonging to the asbestos family).

Chrysotile/asbestiform antigorite was detected (in extremely low quantity) in only one of the examined samples. Overall, crocidolite was the most represented asbestos (51% of the totality of asbestos in all the samples) (Figure 3a), followed by amosite (46%) (Figure 3b), tremolite/actinolite asbestos (3.3%) (Figure 3c), and anthophyllite asbestos (0.9%) (Figure 3d). In the lung samples of subjects with occupational exposure, the median concentrations of crocidolite and amosite were similar, with prevalence of the former.

We did not find any significant differences in the amount of fibers belonging to the five amphibole asbestos species comparing the lung content of individuals with the three types of exposure. In the three exposure groups, the definitely prevalent species were crocidolite and amosite. Smaller quantities of tremolite/actinolite asbestos and anthophyllite asbestos were also found.

### 3.3. Concentration of Asbestos, Other Inorganic Fibers, and ABs and Cause of Death

As previously specified, the subjects of the present study underwent a forensic autopsy because they died with a disease related to asbestos exposure. The subjects who died of MM were 59, whereas 13 out of 72 suffered from asbestosis and died of its complications, such as cardiac-respiratory failure, pneumonia, or other natural causes related to their interstitial lung disease.

Most asbestosis patients were the elderly affected by multiple diseases. However, the most important aspect is that they underwent a documented heavy asbestos exposure, but they did not develop MM. In consideration of their old age and the time elapsed since the beginning of asbestos exposure, it is possible to assume that they would never have developed MM if they had survived longer. Most of the subjects without any ABs or asbestos fibers in lung samples (19.4%) died of MM.

Subjects who died of MM showed a significantly lower median amount of asbestos ff/gdw as compared to subjects who died of other causes (*p* < 0.001) (Table 3).

### 3.4. Concentration of Asbestos Type by Cause of Death and Histological Characteristics of the Neoplasm

Concerning the cause of death, the median concentrations of each type of asbestos showed a tendency to be lower in MM patients compared to subjects without MM, consistent with the significantly lower total amount of asbestos observed in the former group (Table 2). This difference was statistically significant for crocidolite and amosite. On the contrary, the concentration of anthophyllite asbestos and tremolite/actinolite asbestos did not show significant differences in relation to the cause of death or to the type of exposure.

Finally, subjects who died from MM were divided into three groups according to the histological characteristics of the neoplasm: epithelial, sarcomatoid, and biphasic. No statistically significant differences were observed in the total amount of asbestos, as well as in the distribution of each type of asbestos, in relation to the histologic classification of MM.

## 4. Discussion

Interestingly, in the majority of samples taken from MM patients analyzed here, the concentration of asbestos was lower than expected. In 45 out of 59 MM cases (76.3%), the amount of asbestos fibers was found to be well below the threshold value considered demonstrative for occupational exposure to asbestos: more than 0.1 million fibers of amphibole asbestos longer than 5 μm per 1 gdw or more than 10 × 105 fibers of amphibole asbestos longer than 1 μm per 1 gdw as measured by electron microscopy in a qualified laboratory [33]. It is also notable that, in 19.4% subjects who died from MM, neither asbestos nor ABs were detected. This does not mean that the subjects’ lungs were totally free from asbestos—only that we did not find any in our investigation, which involves a limited amount of lung parenchyma. This result indicates that, in those cases, asbestos fibers, if present in lungs, were certainly very few.

In this regard, it is worth pointing out that a low “background” exposure to asbestos is widely diffused. Previously, Capella et al. [31] performed an SEM-EDS analysis of lung samples taken from people who resided in Torino all their life, without any occupational exposure to asbestos (and without anthropogenic environmental exposure, given that in the area of Torino, in that period, there were no plants using asbestos), and died from causes not related to asbestos exposure. In most of these subjects, a low amount of asbestos, from the tremolite/actinolite asbestos and chrysotile/asbestiform antigorite groups, was detected [31]. Likewise, as reported in another paper, non-negligible amounts of asbestos have been found in the general population of Milan [34]. Such data suggest that the general population is potentially exposed to low amounts of asbestos. Yet, evidently, such “background” exposure is not sufficient, in most cases, by itself, to cause MM. The finding of a lower amount of asbestos in lungs of people who died of MM compared to exposed people deceased from other causes corroborates this concept, suggesting that the quantity of asbestos is not decisive in determining MM. In fact, on the other hand, we detected high amounts of asbestos (as well as ABs) in occupationally exposed subjects who never developed MM.

Such findings are consistent with previous studies about the asbestos lung content in which the concentration of amphibole asbestos in MM patients showed no statistically significant differences compared to controls [3]. Other studies found that the MM risk was proportional to the asbestos burden [7] or an increased asbestos concentration in mesothelioma patients [8,9,14,35].

Our findings suggest that MM risk is not directly related to the dose of asbestos in the lungs. Equally important, such results suggest that an extremely small quantity of respired asbestos is sufficient to cause MM. The low doses of asbestos observed in MM cases may be due to previous chrysotile exposure, which could have been cleared from the lungs.

Indeed, the carcinogenic potential of asbestos at very low doses was underlined for the first time by Selikoff’s pioneer study [36]; the author also hypothesized for the first time the possible role of individual susceptibility, perhaps genetically mediated. The present results confirm the absence of a threshold level of asbestos exposure below which there is no risk for MM, as already stated before [36,37,38].

In order to better understand the link between the dose of asbestos exposure and MM risk, electron microscopy analysis of the lung content is necessary. In fact, epidemiological studies often fail to characterize the exposure, as demonstrated by subsequent SEM-EDS studies on the same cohorts [24]. Indeed, despite epidemiological studies suggesting a dose–response relation in the risk of developing MM (e.g., [7,39]), there is no evidence of a relation between asbestos dose and MM risk based on lung inorganic fiber burden studies that provide objective information about the actual inorganic fiber content of the lungs. As demonstrated by electron microscopy, MM often occurs in patients with an asbestos burden comparable to the general population (the so-called “background” or “spontaneous mesothelioma”) [35].

Interestingly, there was a great variability in the amount of ABs, that, albeit statistically correlated with the number of fibers, was extremely variable and often very different from the amount of fibers. In three cases, we observed a concentration of fibers above the threshold of 0.1 million per gdw, but no ABs. On the other hand, in six cases, we detected zero asbestos fibers but a remarkable number of ABs. This result is in line with previous reports that pointed out considerable variability in the efficiency of the coating process from subject to subject [40].

In subjects who died with asbestosis, compared to MM patients, a higher concentration of ABs was observed. Even though this can be related simply to the higher concentration of asbestos fibers in those subjects, resulting in AB formation (consistent with the significant correlation between asbestos fibers and ABs), this finding suggests the hypothesis that the formation of ABs, one of the most controversial and unclear points in the cellular reaction to asbestos, might be the expression of a different biological response to asbestos, leading to a stronger capability to try to isolate and neutralize asbestos [1,41,42]. In other words, this finding may reflect a protective role of the fiber coating in the lung microenvironment.

Moreover, the present study pointed out that the total amount of asbestos, as well as ABs, was not significantly different between subjects with only occupational exposure and those with only anthropogenic environmental exposure. The anamnestic data, which include a detailed residential history, showed that most subjects with anthropogenic environmental exposure used to live very close to the asbestos cement plant (500 m or closer). In six cases, the environmental exposure was both residential and household. The relevance of non-occupational exposure in the determination of the asbestos fiber burden in lungs is in conformity with previous epidemiological studies [23,30,39,43,44,45], as well as with previous electron microscope investigations [14,15]. This finding is in line with the concept that asbestos concentrations in lungs due to anthropogenic environmental exposure can be as high as those provoked by occupational exposure, as stated, as well, by the above cited papers. This means that the environmental exposure to asbestos fibers, nowadays still present worldwide due to the diffusion of asbestos products in many countries, is as effective as occupational exposure in determining the asbestos burden in the lungs.

In addition, a significant difference was found in AB amounts between subjects exposed in both manners and those with only anthropogenic environmental exposure. The concentration of uncoated fibers showed a similar tendency, even though it did not reach statistical significance. AB concentration, though not always proportional to the number of uncoated fibers, is an established marker of asbestos exposure [33,42]. The presented data suggest that the additional effect of the two exposures, when occurring in the same subject, significantly increases the accumulated asbestos fibers in lungs (that, to a certain extent, are coated and detectable as ABs).

The present study also pointed out remarkable findings about the presence of various types of asbestos in lungs. Occupationally exposed subjects were employed at the asbestos cement factory where large amounts of chrysotile and crocidolite were used. Amosite was used in smaller quantities as an additive [19]. Note also that the anthropogenic environmental exposure was related to the production of the same factory, involving people that used to live nearby the plant or whose husband or son used to work in the industry. Notwithstanding, as already explained above, we found almost no fibers attributable with certainty to chrysotile (belonging to the chrysotile/asbestiform antigorite group). Two possible explanations can be proposed.

The first one is obviously related to the well-established rapid clearance of this mineral in the lungs [46,47,48,49]. Despite the intense debate, still ongoing in the literature, the actual biopersistence and the carcinogenic effect of chrysotile are still unanswered questions. From a mineralogical point of view, chrysotile is very different from amphibole asbestos in regard to the chemical composition and structure [50,51]. It is well known that the retention of chrysotile in human lungs is much lower compared to amphiboles due to its rapid clearance [52]. The mechanism of chrysotile elimination from lungs, though not fully understood, is related to the fragmentation of the fibers in the lung microenvironment and the subsequent phagocytosis by macrophages (airway macrophages, alveolar macrophages, interstitial macrophages, intravascular macrophages, and pleural macrophages) [46]. The chemical instability of chrysotile is due to the dissociation of magnesium atoms from the crystalline structure in the acid lung microenvironment; as a consequence, the structure of chrysotile becomes friable and fragments into very small pieces of fibrils that can be phagocytized and removed from the alveoli [50]. Recent experimental studies on rats and baboons confirmed a very rapid clearance of chrysotile from lungs (with very few fibers after 90 days since the end of exposure, compared to high concentration of amphiboles after the same period of time) [53,54]. However, other studies showed findings that imply the opposite deductions. For instance, a recent paper by Feder and colleagues showed that asbestos, in particular, chrysotile, is stable in human lungs for up to 37 years [25], and chrysotile is the main fiber they observed in human lung samples using a high-resolution electron microscope, a FEG-SEM. Similarly, previous studies on animals, as well as on humans, pointed out the presence of chrysotile as late as 60 years after exposure [35]. Churg and De Paoli, in 1988 [47], measuring the fiber burden in lungs of subjects with different time intervals since exposure cessation, concluded that respired chrysotile may meet two different fates: part of the fibers is cleared quickly from the air spaces; the other part manages to reach the interstitium and remains for longer. Such observations suggested that the degradation of chrysotile in human lungs, leading to its clearance, must occur very early since inhalation and after that, the remaining chrysotile, which does not degrade in a short time, is not significantly cleared in the following years.

The data presented here instead suggest a complete degradation and removal of chrysotile in all investigated subjects except one (who had very few chrysotile fibers in his lungs). In our series, the time intervals since the cessation of the exposure, occupational or anthropogenic environmental, were extremely inhomogeneous (range: 8–44 years), but we did not notice any difference in chrysotile presence, suggesting that the clearance of this mineral occurs relatively rapidly, consistent with what is already known [48,52].

Another possible explanation should be considered: chrysotile fibers might be too thin to be detected using the technique described above (SEM-EDS observation using the mentioned instrumentation), due to a resolution power that was limited to 0.2 um. Indeed, in the study by Feder et al. [25], a FEG-SEM was used, with a higher resolution: under such conditions, chrysotile was detected. Nevertheless, studies on the fiber burden in lungs measured by TEM (that has a higher resolution than SEM) found very few chrysotile fibers compared to amphibole asbestos [7,16], even in subjects known to be exposed to chrysotile. Such results, in line with the data presented here, support the hypothesis of the preponderant role of the pulmonary degradation of chrysotile.

The predominance of crocidolite, followed by amosite, in the lungs of almost all subjects is consistent with the production at the plant [17,19] and the durability of this kind of asbestos. Moreover, in subjects with anthropogenic environmental exposure in Broni, compared to those who were occupationally exposed, a higher proportion of tremolite/actinolite asbestos was observed (albeit not statistically significant). Tremolite/actinolite asbestos was never used commercially, but it is dispersed from natural sources from the outcropping rocks containing them (serpentinite rocks in the Western Alps) and from talc-containing materials, when the talc contains these types of asbestos as natural contaminants [31] or, less frequently, anthophyllite asbestos [55]. Therefore, their air dispersion could be due to the use of talc or talc-containing products, given the large industrial use of talc [56].

The different carcinogenic potential of chrysotile and tremolite/actinolite asbestos is still questioned. It was suggested that chrysotile-induced MM in miners (exposed to mine dust) is indeed related to the presence of tremolite/actinolite asbestos in the mineral ore before milling, therefore separating chrysotile from tremolite/actinolite asbestos [10,57]. Our observations suggest a high carcinogenic potential of tremolite/actinolite asbestos, even though its association with chrysotile is not clear.

When exposed both occupationally and environmentally, subjects showed a prevalence of amosite, whereas in the groups only occupationally exposed, crocidolite and amosite were similarly distributed. Interestingly, subjects with anthropogenic environmental exposure showed a different distribution compared to occupational exposure: tremolite/actinolite asbestos was represented similarly to amosite, and there was a presence of anthophyllite asbestos that was not used at the plant (and, in fact, was not detected in the lungs of occupationally exposed subjects). This may suggest other sources of exposure (such as utensils or other products containing asbestos that were widely used at the time).

Concerning the differences in the various types of asbestos according to the cause of death (MM or not), the present investigations pointed out that subjects who died from MM not only had a lower amount of total asbestos, but specifically less crocidolite and amosite, considered the most noxious kind of asbestos (according to their different biopersistences) [51,58,59]. This might be related to the fact that all subjects who were heavily exposed to asbestos and died from causes other than MM were employed at the asbestos cement factory, where amosite and crocidolite were used. In fact, they had high concentrations of such asbestos types in their lungs. However, crocidolite and amosite have been detected in most MM cases (though in low amounts). On the contrary, previous studies have reported an absence of these kinds of asbestos in the general population [31,34]. This suggests that MM onset is likely to be related to crocidolite and amosite, albeit in small quantities. Yet, given that even more crocidolite and amosite were detected in lungs of subjects who were exposed to asbestos but did not die of MM, it seems obvious that the presence of such types in the lungs is not sufficient, even in large amounts, to cause MM.

## 5. Conclusions

The results presented here are the outcome of a multidisciplinary study, involving different scientific fields (legal medicine, pathology, occupational medicine, and environmental mineralogy), and have relevant implications from a prevention point of view, corroborating the hypothesis that the amount of asbestos is not determinant for MM risk. In fact, we found high quantities of asbestos in heavily exposed subjects who never developed MM. On the other hand, numerous subjects who died of MM showed an asbestos lung content comparable to the general population and, in a remarkable proportion of them, no asbestos was detected. These results confirm that the risk of MM is not related to asbestos dose, as opposed to asbestosis, which is clearly dose-dependent.

Equally important, our findings call for more research on the biopersistence of asbestos in the lung microenvironment and on the individual differences in the biological response to asbestos inhalation and respiration (especially in relation to the formation of ABs).

## Figures and Tables

**Table 1:** Demographic and significant anamnestic data of the 72 subjects analyzed in this study.

Demographic/Anamnestic Characteristics	*n* = 72
Sex	
male	62 (86.1%)
female	10 (13.9%)
Cause of death	
no MM	13 (18.1%)
MM	59 (81.9%)
Histological type of MM	
epithelial	38 (65.5%)
sarcomatoid	6 (10.3%)
biphasic	14 (24.1%)
Type of exposure	
both exposures	26 (36.1%)
environmental exposure	26 (36.1%)
occupational exposure	20 (27.8%)
Latency (occupational exposure), years	median = 41.0 (IQR * 33.0–48.0)
Latency (environmental exposure), years	median = 53.0 (IQR 42.0–65.0)
Exposure duration (occupational), months	median = 264.0 (IQR 108.0–360.0)
Exposure duration (environmental), months	median = 414.0 (IQR 258.0–576.0)
Survival time since diagnosis of MM, months	median = 15.0 (IQR 9.5–28.5)
Time since end of exposure, years	median = 21.0 (IQR 18.0–26.0)

* IQR= Interquartile Range.

**Table 2:** Amount of asbestos fibers and ABs (Asbestos Bodies) in the lungs, counted and classified using SEM-EDS: comparison between subjects with occupational exposure alone, anthropogenic environmental exposure alone, and both exposures.

Asbestos Fibers and ABs	Occupational Exposure Alone (*n* = 20)	Anthropogenic Environmental Exposure Alone (*n* = 26)	Both Exposures (*n* = 26)	Test * and *p*-Value
**Asbestos per** **ff/gdw** **Median** (IQR **)	22,530.5 (4426.2–267,890.8)	20,336.9 (0.0–65,623.0)	24,199.7 (0.0–297,895.0)	KW= 3.30 0.192
**ABs per** **ff/gdw** **Median** **(IQR)**	6292.0 (0.0–364,735.6)	0.0 (0.0–19,985.9)	34,002.4 (3369.0–353,750.0)	KW = 9.85 0.007

* Kruskall–Wallis test = KW. ** IQR= Interquartile Range.

**Table 3:** Amount of asbestos fibers, ABs (Asbestos Bodies), and each asbestos type in lungs, counted and classified using SEM-EDS: comparison between subjects who died of MM (Mesothelioma) and those who died of other causes (No mesothelioma).

Fibers, Abs, Asbestos Type	No Mesothelioma	Mesothelioma	Test *
	(*n* = 13)	(*n* = 59)	and *p*-Value
**Asbestos fibers per ff/gdw**			
**Median**	297,895.0	11,320.0	MW = 3.71
(IQR **)	(30,321.4–881,567.5)	(0.0–92,282.6)	<0.001
**ABs per ff/gdw**			
**Median**	452,800.0	4579.3	MW = 1.97
**(IQR)**	(0.0–664,502.8)	(0.0–50,535.7)	0.0
**Chrysotile/asbestiform Antigorite**			
**Median**	0.0	0.0	MW = 2.13
**(IQR)**	(0–0)	(0–0)	0.0
**Crocidolite**			
**Median**	141,450.0	0.0	MW = 4.23
**(IQR)**	(70,750.0–348,134.9)	(0.0–28,605.1)	<0.001
**Amosite**			
**Median**	178,736.8	0.0	MW = 3.50
**(IQR)**	(15,160.7–516,587.3)	(0.0–28,605.1)	<0.001
**Anthophyllite asbestos**			
**Median**	0.0	0.0	MW = 0.21
**(IQR)**	(0.0–0.0)	(0.0–0.0)	0.8
**Tremolite/actinolite asbestos**			
**Median**	0.0	0.0	MW = 1.70
**(IQR)**	(0.0–5660.0)	(0.0–9158.6)	0.1

* Mann–Whitney test = MW. ** IQR= Interquartile Range.

## Data Availability

The data presented in this study are available on request from the corresponding author. The data are not publicly available due to privacy and ethical restrictions.

## References

[1] Gaudino G., Xue J., Yang H. (2020). How asbestos and other fibers cause mesothelioma. Transl. Lung. Cancer Res..

[2] Carbone M., Adusumilli P.S., Alexander H.R., Baas P., Bardelli F., Bononi A., Bueno R., Felley-Bosco E., Galateau-Salle F., Jablons D. (2019). Mesothelioma: Scientific clues for prevention, diagnosis, and therapy. CA Cancer J. Clin..

[3] Wagner J.C., Berry G., Pooley F.D. (1982). Mesotheliomas and asbestos type in asbestos textile workers: A study of lung contents. Br. Med. J..

[4] Churg A., Wiggs B., Depaoli L., Kampe B., Stevens B. (1984). Lung asbestos content in chrysotile workers with mesothelioma. Am. Rev. Respir. Dis..

[5] McDonald J.C., Armstrong B., Case B., Doell D., McCaughey W.T., McDonald A.D., Sébastien P. (1989). Mesothelioma and asbestos fiber type. Evidence from lung tissue analyses. Cancer.

[6] Churg A., Vedal S. (1994). Fiber burden and patterns of asbestos-related disease in workers with heavy mixed amosite and chrysotile exposure. Am. J. Respir. Crit. Care Med..

[7] Gilham C., Rake C., Burdett G., Nicholson A.G., Davison L., Franchini A., Carpenter J., Hodgson J., Darnton A., Peto J. (2016). Pleural mesothelioma and lung cancer risks in relation to occupational history and asbestos lung burden. Occup. Environ. Med..

[8] Sakai K., Hisanaga N., Huang J., Shibata E., Ono Y., Aoki T., Takagi H., Ando T., Yokoi T., Takeuchi Y. (1994). Asbestos and nonasbestos fiber content in lung tissue of Japanese patients with malignant mesothelioma. Cancer.

[9] Rogers A.J., Leigh J., Berry G., Ferguson D.A., Mulder H.B., Ackad M. (1991). Relationship between lung asbestos fiber type and concentration and relative risk of mesothelioma. A case-control study. Cancer.

[10] Gibbs G.W., Berry G. (2008). Mesothelioma and asbestos. Regul. Toxicol. Pharmacol..

[11] Roggli V.L., Pratt P.C., Brody A.R. (1993). Asbestos fiber type in malignant mesothelioma: An analytical scanning electron microscopic study of 94 cases. Am. J. Ind. Med..

[12] Morinaga K., Kohyama N., Yokoyama K., Yasui Y., Hara I., Sasaki M., Suzuki Y., Sera Y. (1989). Asbestos fibre content of lungs with mesotheliomas in Osaka, Japan: A prelihary report. IARC Sci. Publ..

[13] Friedrichs K.H., Brockmann M., Fischer M., Wick G. (1992). Electron microscopy analysis of mineral fibers in human lung tissue. Am. J. Ind. Med..

[14] Barbieri P.G., Mirabelli D., Somigliana A., Cavone D., Merler E. (2012). Asbestos fibre burden in the lungs of patients with mesothelioma who lived near asbestos cement factories. Ann. Occup. Hyg..

[15] Barbieri P.G., Somigliana A., Chen Y., Consonni D., Vignola R., Finotto L. (2020). Lung Asbestos Fibre Burden and Pleural Mesothelioma in Women with Non-occupational Exposure. Ann. Work. Expo. Health.

[16] Magnani C., Mollo F., Paoletti L., Bellis D., Bernardi P., Betta P., Botta M., Falchi M., Ivaldi C., Pavesi M. (1998). Asbestos lung burden and asbestosis after occupational and environmental exposure in an asbestos cement manufacturing area: A necropsy study. Occup. Environ. Med..

[17] Oddone E., Ferrante D., Cena T., Tùnesi S., Amendola P., Magnani C. (2014). Asbestos cement factory in Broni (Pavia, Italy): A mortality study. Med. Lav..

[18] Mensi C., Riboldi L., De Matteis S., Bertazzi P.A., Consonni D. (2015). Impact of an asbestos cement factory on mesothelioma incidence: Global assessment of effects of occupational, familial, and environmental exposure. Environ. Int..

[19] Oddone E., Ferrante D., Tunesi S., Magnani C. (2017). Mortality in asbestos cement workers in Pavia, Italy: A cohort study. Am. J. Ind. Med..

[20] Binazzi A., Zona A., Marinaccio A., Bruno C., Corfiati M., Fazzo L., Menegozzo S., Nicita C., Pasetto R., Pirastu R. (2016). SENTIERI-ReNaM: Results. Epidemiol. Prev..

[21] Mensi C., De Matteis S., Dallari B., Riboldi L., Bertazzi P.A., Consonni D. (2016). Incidence of mesothelioma in Lombardy, Italy: Exposure to asbestos, time patterns and future projections. Occup. Environ. Med..

[22] Visonà S.D., Villani S., Manzoni F., Chen Y., Ardissino G., Russo F., Moretti M., Javan G.T., Osculati A. (2018). Impact of asbestos on public health: A retrospective study on a series of subjects with occupational and non-occupational exposure to asbestos during the activity of Fibronit plant (Broni, Italy). J. Public Health Res..

[23] Consonni D., De Matteis S., Dallari B., Pesatori A.C., Riboldi L., Mensi C. (2020). Impact of an asbestos cement factory on mesothelioma incidence in a community in Italy. Environ. Res..

[24] Roggli V.L. (2016). Fiber analysis vignettes: Electron microscopy to the rescue!. Ultrastruct. Pathol..

[25] Feder I.S., Tischoff I., Theile A., Schmitz I., Merget R., Tannapfel A. (2017). The asbestos fibre burden in human lungs: New insights into the chrysotile debate. Eur. Respir. J..

[26] Belluso E., Bellis D., Fornero E., Capella S., Ferraris G., Coverlizza S. (2006). Assessment of Inorganic Fibre Burden in Biological Samples by Scanning Electron Microscopy—Energy Dispersive Spectroscopy. Microchim. Acta.

[27] World Health Organization (2000). Regional Office for Europe Air Quality Guidelines for Europe.

[28] De Vuyst P., Karjalainen A., Dumortier P., Pairon J.C., Monsó E., Brochard P., Teschler H., Tossavainen A., Gibbs A. (1998). Guidelines for mineral fibre analyses in biological samples: Report of the ERS Working Group. European Respiratory Society. Eur. Respir. J..

[29] Gunter M.E., Belluso E., Mottana A., Rosso J.J. (2007). Amphiboles: Crystal Chemistry, Occurrence, and Health Issues. Reviews in Mineralogy and Geochemistry.

[30] Marinaccio A., Binazzi A., Bonafede M., Corfiati M., Di Marzio D., Scarselli A., Verardo M., Mirabelli D., Gennaro V., Mensi C. (2015). Malignant mesothelioma due to non-occupational asbestos exposure from the Italian national surveillance system (ReNaM): Epidemiology and public health issues. Occup. Environ. Med..

[31] Capella S., Bellis D., Fioretti E., Marinelli R., Belluso E. (2020). Respirable inorganic fibers dispersed in air and settled in human lung samples: Assessment of their nature, source, and concentration in a NW Italy large city. Environ. Pollut..

[32] Langevin S.M., Kelsey K.T., Anttila S., Boffetta P. (2020). Mechanisms of Environmental and Occupational Carcinogenesis. Occupational Cancers.

[33] Wolff H., Vehmas T., Oksa P., Rantanen J., Vainio H. (2015). Asbestos, asbestosis, and cancer, the Helsinki criteria for diagnosis and attribution 2014: Recommendations. Scand. J. Work Environ. Health.

[34] Casali M., Carugno M., Cattaneo A., Consonni D., Mensi C., Genovese U., Cavallo D.M., Somigliana A., Pesatori A.C. (2015). Asbestos Lung Burden in Necroscopic Samples from the General Population of Milan, Italy. Ann. Occup. Hyg..

[35] Neumann V., Löseke S., Tannapfel A. (2011). Mesothelioma and analysis of tissue fiber content. Recent Results Cancer Res..

[36] Selikoff I.J., Lee D.H.K. (1978). Asbestos and Disease.

[37] Tomatis L., Cantoni S., Carnevale F., Merler E., Mollo F., Ricci P., Silvestri S., Vineis P., Terracini B. (2007). The role of asbestos fiber dimensions in the prevention of mesothelioma. Int. J. Occup. Environ. Health.

[38] Hodgson J.T., Darnton A. (2000). The quantitative risks of mesothelioma and lung cancer in relation to asbestos exposure. Ann. Occup. Hyg..

[39] Bourdès V., Boffetta P., Pisani P. (2000). Environmental exposure to asbestos and risk of pleural mesothelioma: Review and meta-analysis. Eur. J. Epidemiol..

[40] Morgan A., Holmes A. (1980). Concentrations and dimensions of coated and uncoated asbestos fibres in the human lung. Br. J. Ind. Med..

[41] Crovella S., Bianco A.M., Vuch J., Zupin L., Moura R.R., Trevisan E., Schneider M., Brollo A., Nicastro E.M., Cosenzi A. (2016). Iron signature in asbestos-induced malignant pleural mesothelioma: A population-based autopsy study. J. Toxicol. Environ. Health A.

[42] Dodson R.F., Williams M.G., O’Sullivan M.F., Corn C.J., Greenberg S.D., Hurst G.A. (1985). A comparison of the ferruginous body and uncoated fiber content in the lungs of former asbestos workers. Am. Rev. Respir. Dis..

[43] Kurumatani N., Kumagai S. (2008). Mapping the risk of mesothelioma due to neighborhood asbestos exposure. Am. J. Respir. Crit. Care Med..

[44] Maule M.M., Magnani C., Dalmasso P., Mirabelli D., Merletti F., Biggeri A. (2007). Modeling mesothelioma risk associated with environmental asbestos exposure. Environ. Health Perspect..

[45] Marsh G.M., Riordan A.S., Keeton K.A., Benson S.M. (2017). Non-occupational exposure to asbestos and risk of pleural mesothelioma: Review and meta-analysis. Occup. Environ. Med..

[46] Oberdörster G. (1994). Macrophage-associated responses to chrysotile. Ann. Occup. Hyg..

[47] Churg A., DePaoli L. (1988). Clearance of chrysotile asbestos from human lung. Exp. Lung Res..

[48] Churg A., Wright J.L. (1994). Persistence of natural mineral fibers in human lungs: An overview. Environ. Health Perspect..

[49] Finkelstein M.M., Dufresne A. (1999). Inferences on the kinetics of asbestos deposition and clearance among chrysotile miners and millers. Am. J. Ind. Med..

[50] Bernstein D., Dunnigan J., Hesterberg T., Brown R., Velasco J.A.L., Barrera R., Hoskins J., Gibbs A. (2013). Health risk of chrysotile revisited. Crit. Rev. Toxicol..

[51] Bernstein D.M. (2014). The health risk of chrysotile asbestos. Curr. Opin. Pulm. Med..

[52] Churg A. (1994). Deposition and clearance of chrysotile asbestos. Ann. Occup. Hyg..

[53] Bernstein D.M., Toth B., Rogers R.A., Kling D.E., Kunzendorf P., Phillips J.I., Ernst H. (2020). Evaluation of the exposure, dose-response and fate in the lung and pleura of chrysotile-containing brake dust compared to TiO2, chrysotile, crocidolite or amosite asbestos in a 90-day quantitative inhalation toxicology study—Interim results Part 1: Experimental design, aerosol exposure, lung burdens and BAL. Toxicol. Appl. Pharmacol..

[54] Rendall R.E.G., Du Toit R.S.J. (1994). The Retention and Clearance of Glass Fibre and Different Varieties of Asbestos by the Lung. Ann. Occup. Hyg..

[55] Gunter M.E., Buzon M.E., McNamee B.D. (2018). Current issues with purported “asbestos” content of talc: Asbestos nomenclature and examples in metamorphic carbonate and ultramafic hosted talc ores. Trans. Soc. Min. Metall. Explor..

[56] Talc and Pyrophyllite Statistics and Information. https://www.usgs.gov/centers/nmic/talc-and-pyrophyllite-statistics-and-information.

[57] Churg A. (1988). Chrysotile, tremolite, and malignant mesothelioma in man. Chest.

[58] Hobbs C.H. Fiber Durability and Biopersistence—Assessment and Role in Asbestos Toxicology. Proceedings of the EPA’s Asbestos Mechanisms of Toxicity Workshop.

[59] Berman D.W., Crump K.S. (2008). A meta-analysis of asbestos-related cancer risk that addresses fiber size and mineral type. Crit. Rev. Toxicol..

